# Neurocognitive function in schizophrenia spectrum disorders: A 20-year prospective study of a community sample

**DOI:** 10.1016/j.scog.2025.100393

**Published:** 2025-09-30

**Authors:** Christine Mohn, Anna-Karin Olsson, Maivor Olsson-Tall, Fredrik Hjärthag, Iris van Dijk Härd, Lars Helldin

**Affiliations:** a National Centre for Suicide Research and Prevention, Institute of Clinical Medicine, University of Oslo, Oslo, Norway; b Department of Psychiatry, NU Health Care Hospital, Trollhättan, Västra Götaland Region, Sweden; c Department of Psychology, Karlstad University, Karlstad, Sweden; d Institute of Health Sciences, University West, Trollhättan, Sweden

**Keywords:** Longitudinal study, Memory, Neurocognition, Prospective study, Psychosis, Schizophrenia

## Abstract

Longitudinal studies of neurocognition in schizophrenia spectrum disorders (SSD) usually follow relatively young first-episode patients across several years. Comparatively little is known about the neurocognitive trajectory of samples also consisting of older patients. This is a 20-year follow-up study of participants who performed the baseline assessment at different ages and utilizes data from the Swedish Clinical Long-Term Psychosis Study (CLIPS). At baseline, 61 SSD patients were included and available for clinical assessment after 20 years. Of these, 28 performed neurocognitive assessment at both baseline and 20 years later. The test results from this group were used for this study. After 20 years, the participants exhibited significantly worsening cognitive flexibility, verbal learning, verbal retention memory, and verbal intellectual function compared to baseline. All the statistically significant differences from baseline to follow-up had large effect sizes. The other cognitive domains showed no statistically significant changes from baseline for either group. We conclude that although the overall picture was one of neurocognitive stability across 20 years, our participants showed signs of accelerated ageing in the verbal domain specifically.

## Highlights

• We studied Swedish schizophrenia spectrum disorder patients.
• Their neurocognitive function was assessed at baseline and 20 years later.
• We found reduced verbal learning, verbal memory, and verbal IQ.
• The other neurocognitive domains showed stability across time.
• We suggest this indicates accelerated cognitive ageing in the verbal domain.

## 1 Introduction

Neurocognitive impairment is a core feature of schizophrenia spectrum disorders (SSD) (Stone et al., 2022). It is evident across the entire illness spectrum from the prodromal phase (Mollon and Reichenberg, 2018) through the first episode in young adulthood (Torgalsbøen et al., 2023) and middle age (Cuesta et al., 2022). The cognitive deficits tend to persist even if psychosis symptoms, e.g., hallucinations and delusions, may recede (Rund et al., 2016).

The long-term nature of neurocognitive impairment in SSD is still a matter of debate. It is not certain if the neurocognitive deficits of SSD patients remain stable throughout life, or if they deteriorate in older adulthood. A recent meta-analysis found no significant decline or improvement in neurocognitive function during the first 5 years after illness onset (Watson et al., 2022). Some studies that have followed SSD patients for 10 years or more also point to cognitive stability. In the Norwegian TIPS study of first-episode SSD patients, the general neurocognitive composite score was stable across 10 years (Rund et al., 2016). In a smaller Norwegian sample of young first-episode SSD patients, there was a significant improvement in processing speed, attention, visual learning, executive function, and social cognition during the first 6 years of the 10-year follow-up period, and then cognitive stability (Torgalsbøen et al., 2023). Flaaten et al. (2022) using 10-year follow-up data from the Norwegian TOP project observed heterogeneity within the first-episode SSD sample, in that the majority exhibited stable neurocognitive function while smaller subgroups deteriorated or improved. In a study from the British AESOP project, Zanelli et al. (2019) followed fist-episode SSD patients and healthy controls for 10 years, observing stability in most of the cognitive domains. However, there was a specific decline in IQ, verbal memory and verbal knowledge in the patient group. Later, the same research group pinpointed the age of 40 as the beginning of the decline in verbal memory in the SSD group (Zanelli et al., 2022). A cross-sectional US study of neurocognitive function in first-episode patients across different age brackets also noted differences in the groups younger and older than 40 years (Mollon et al., 2020).

Fett et al. (2020) followed the US Suffolk County cohort of first-episode SSD patients and healthy controls for 20 years and found a sharper decline in processing speed, attention, verbal and visual memory, and executive functions in the patient group compared to the control group. In a 25-year follow-up assessment of the same cohort, Jonas et al. (2022) confirmed the Fett et al. (2020) results, while also detecting an aggravated decline beginning at 22 years after illness onset.

While the above studies (Fett et al., 2020; Jonas et al., 2022; Zanelli et al., 2019, Zanelli et al., 2022) support the notion of accelerated cognitive ageing in SSD patients in general, there may be heterogeneous trajectories in different subgroups of patients. Starzer et al. (2024) performed a 20-year follow-up assessment of the first-episode cohort of the Danish OPUS study and identified three cognitive trajectories – one group showed decline (30 %), one group showed stability (49 %), and one group showed improved function (20 %).

All of the above studies have investigated first-episode schizophrenia patients, most of whom were older teenagers or young adults at baseline. Consequently, most of them were middle aged at endline. Including patients at different stages of illness and age at baseline would provide a more representative picture of the SSD population. This is particularly important in order to identify the presence of accelerated cognitive ageing, as patients exhibiting this characteristic would be in need of extra care.

This paper presents data from the Clinical Long-Term Psychosis Study (CLIPS) in Western Sweden, in which community-dwelling SSD patients are followed for 20 years in terms of clinical, neurocognitive, and real-world function. A feature of this project is that participants were included at various ages, illness stages, and number of previous treatment contacts. Therefore, we have the unique opportunity to study long-term neurocognitive function also in older SSD patients, which is a group of people that is often neglected in this type of research.

We have previously reported baseline neurocognitive impairment in the patients (Karilampi et al., 2011), and that this impairment is related to unstable long-term remission (Johansson et al., 2020) and excessive mortality (Mohn et al., 2023). Here, we investigate our sample's neurocognitive function across 20 years aiming to determine which domains improve, decline, or remain stable.

## 2 Methods

### 2.1 Participants

This study is part of the CLIPS project, which has been described in detail elsewhere (e.g., Helldin et al., 2015). Briefly, men and women suffering from SSD were recruited from the outpatient clinics in the NU Health Hospital region in South-Western Sweden from November 2000. Two thirds of all eligible patients on this geographic catchment area were included at baseline. The participants were required to be in a stable clinical condition. Exclusion criteria were age below 18, mental retardation precluding the ability to give informed consent, past or present neurological illness, and significant past or present use of alcohol or illegal substances. Each participant gave their written informed consent to participation. The project was approved by the Research Ethics Committee in Gothenburg, Sweden (# Ö537–99, 507–04, 438–10, 423–14, 811–16) and carried out according to the Helsinki Declaration.

Of the 501 participants included at baseline, 61 were available for clinical reassessment 20 years later. All assessments were performed in Swedish. One participant was bilingual, but did not require the assistance of an interpreter. The sub-diagnoses at baseline were schizophrenia *n* = 42 (68.9 %), schizoaffective disorder *n* = 14 (23.0 %), and delusional disorder *n* = 5 (8.2 %).

Not all the participants had completed the full test battery at both time points, and for the neurocognitive analyses, we report data from those where we have matched pairs (baseline and 20 years) of test results (*N* = 28) (Table 3). For these 28 participants, the sub-diagnoses at baseline were schizophrenia *n* = 20 (71.4 %), schizoaffective disorder *n* = 6 (21.4 %), and delusional disorder n = 2 (7.1 %). In order to present as much information about our participants as possible, several tables are provided: Table 1, Table 2 contain demographic and clinical data from the 28 participants for which we have matched pairs neurocognitive data. Supplementary Tables 1 and 2 contain data from all 61 participants. Supplementary Table 3 contains demographic and clinical data for the participants who either completed the neurocognitive assessment at baseline (*N* = 20) or at 20 years (*N* = 13), and who are not part of the 28 participants with matched pairs data.

**Table 1:** Demographic and clinical information (*N* = 28).

	Baseline	20 years
Sex	20 males (71.4) 8 females (28.6)	
Age at inclusion in study, years	41.5 (10.9), range 21–61	
Age at first hospitalization, years	30.3 (11.5), range 17–73	
Duration of treated illness, years	12.5 (10.3), range 0–38	
In remission	16 (57.1)	22 (78.6)^a^
PANSS		
Positive		11.0 (3.9)
Negative		12.2 (5.0)
General		22.0 (5.1)
GAF		
Symptoms	46.5 (6.5)	58.0 (18.1)
Function	51.1 (6.6)	58.3 (14.3)
Antipsychotic medication^b^		
Risperdal Consta		3 (10.7)
Risperdal		2 (7.1)
Clozapine		3 (10.7)
Quetiapine		1 (3.6)
Olanzapine		2 (7.1)
Aripiprazole		5 (17.9)
Paliperidone		1 (3.6)
Ziprasidone		1 (3.6)

Numbers in mean (SD) or N (%). *n* = 10 (35.7 %).

a Missing data *n* = 3 (10.7 %).

b Missing data.

**Table 2:** Social and economic information (N = 28).

	Baseline	20 years
Marital status		
Single (never partnered)	13 (46.4)	15 (53.6)
Divorced/widowed	6 (21.4)	1 (3.6)
Married/partnered	8 (28.6)	8 (28.8)
Missing data	1 (3.6)	4 (14.3)
Housing status		
Long-term hospital/care	1 (3.6)	0
Assisted living facility	0	2 (7.1)
Living with parents	2 (7.1)	1 (3.6)
Own residence	24 (85.0)	22 (78.6)
Missing data	1 (3.6)	3 (10.7)
Education level^a^		
Elementary school	3 (10.7)	
High school	15 (53.6)	
College level	10 (35.7)	
Missing data	0	
Employment status^a^		
Full-time work	12 (42.9)	
Part-time work	1 (3.6)	
Student	3 (10.7)	
Volunteer work	0	
Supported employment	0	
Age/disability pension	0	
Never worked/unknown	1 (3.6)	
Missing data	11 (39.3)	

Numbers in N (%).

a Highest level of education or employment before illness onset.

### 2.2 Assessments

Diagnosis was determined according to the DSM-IV and ISD-10 criteria (APA, 1994; WHO, 1993). Positive, negative, and general symptoms of schizophrenia were assessed with the Positive and Negative Syndrome Scale (PANSS; Kay et al., 1987) at 20-years follow-up. The PANSS was gradually introduced in the CLIPS project, therefore, we have insufficient scores from the baseline assessment and are unable to report them here. Positive Functional level was determined according to the Global Assessment of Function (GAF), and the total score was split into a symptom and function sub score (Pedersen et al., 2007). Remission status was established according to the guidelines of Andreasen et al. (2005). Most participants were treated with antipsychotic medication across the study period, but medication data were available only at endline (Table 1, Supplementary Table 1).

After the clinical assessments, the participants performed the following neurocognitive tests in their Swedish versions:

The *Trail Making Test A (TMT-A) and B (TMT-B)* (Reitan, 1958) were used as measures of visuomotor speed (part A) and cognitive flexibility (part B).
The *Letter Number Span test (LNS)* (Gold et al., 1997) was used as a measure of auditory working memory.
The *Rey Auditory Verbal Learning Test (RAVLT)* (Schmidt, 1996) consists of a 15-item word list that is read aloud to the participant five times. We used the number of words correctly recalled in trial 1 (RAVLT 1) as an indication of immediate memory, trial 1 to trial 5 (RAVLT 1–5 sum) as an indication of learning, and the number of words correctly recalled after 20 min (RAVLT 7) as an indication of retention memory.
The *Wisconsin Card Sorting Test (WCST)* (Heaton et al., 1993) is a computerized test of executive function. We used the 6-category WCST version, with the number of trials, total errors, perseverative responses, perseverative errors, and completed categories as measures of executive function.
The *Vocabulary* subtest from the revised Wechsler Adult Intelligence Scale (WAIS) (Wechsler, 1997) was used as an estimate of pre-morbid crystallized intelligence.

As there are no longitudinal norms for these tests, we report all results in raw scores. Table 3 presents neurocognitive test data from the *N* = 28 matched pairs participants, and Supplementary Table 4 contains data from the participants for which we have either baseline (*N* = 20) or endline (*N* = 13) test results.

**Table 3:** Neurocognitive function in raw scores at baseline and 20-year follow-up (N = 28).

Cognitive domain	Baseline	20 years	*t*-Test	Cohen's d
*Processing speed*				
TMT-A	42.9 (21.5)	50.7 (24.2)	−1.99, p .057	−0.38
*Cognitive flexibility*				
TMT-B	120.7 (114.9)	156.4 (102.2)	−2.59, p .016⁎	−0.50
*Immediate memory*				
RAVLT-1	5.2 (2.1)	4.5 (2.0)	1.49, p .147	0.28
*Short-term memory*				
RAVLT 1–5 sum	44.1 (10.1)	34.1 (12.8)	4.12, *p* < .001⁎⁎⁎	0.79
*Retention memory*				
RAVLT-7	8.9 (3.5)	5.8 (4.0)	4.87, p < .001⁎⁎⁎	0.93
*Working memory*				
LNS	9.6 (2.5)	9.8 (5.4)	−0.21, p .832	−0.04
*Executive function*				
WCST Trials	117.9 (20.8)	115.5 (23.0)	0.44, p .665	0.09
WCST Total errors	53.9 (26.6)	58.0 (27.9)	−0.67, p .509	−0.13
WCST Perseverative responses	33.7 (25.5)	36.3 (25.2)	−0.40, p .694	−0.08
WCST Perseverative errors	28.1 (20.3)	30.3 (19.3)	−0.43, p .673	−0.08
WCST Completed categories	2.6 (2.1)	2.4 (2.3)	0.42, p .682	0.08
*Crystallized intelligence*				
WAIS Vocabulary	39.6 (11.6)	34.4 (12.6)	3.00, p .006⁎⁎	0.58

Numbers in mean (SD). Paired samples t-tests, 2-tailed.

⁎ *p* < .05.

⁎⁎ *p* < .01.

⁎⁎⁎ *p* < .001.

### 2.3 Statistics

All analyses were carried out using IBM SPSS version 29. Differences over time in neurocognitive test scores were analyzed with paired samples *t*-tests with Cohen's d as an estimate of effect size.

## 3 Results

When comparing the neurocognitive scores from baseline to 20-years follow-up, we found a statistically significant increase in the TMT-B score, indicating a worsening of cognitive flexibility. Moreover, there were statistically significant decreases in the scores of RAVLT 1–5 and RAVLT 7, indicating reduced verbal learning and retention memory. Finally, the Vocabulary test, indexing verbal crystallized intelligence, deteriorated significantly across time. The effect sizes of these differences were large (Table 3).

Although no statistical analyses are performed with the sub-samples that completed the neurocognitive assessment at baseline or endline only, visual inspection of Supplementary Table 4 suggests a similar overall picture of function as for the full matched pairs sample.

## 4 Discussion

In this prospective, 20-year follow-up study of a Swedish SSD community sample that was assessed with a comprehensive neurocognitive test battery, we found that most tests scores remained stable and that specific domains, most notably the verbal functions, deteriorated.

The TMT-B score, assessing cognitive flexibility, was reduced across time. This is congruent with the results of another 20-year study (Fett et al., 2020) but in contrast to the 10-year follow-up study of Zanelli et al. (2019), who found no TMT-B changes. The TMT-B test is regarded as corresponding to the WCST perseverative errors subtest in measuring cognitive flexibility (Kortte et al., 2002), and the lack of changes in the WCST test scores in our participants is therefore somewhat surprising. However, executive functions are complex and consisting of several sub-functions and tasks. Therefore, different neuropsychological tests assessing them will most likely not generate completely overlapping results.

Moreover, we observed significant deterioration in the RAVLT 1–5 sum score, assessing verbal learning, and RAVLT 7, assessing retention memory, across 20 years. Again, this aligns with what others have found in 10-year (Zanelli et al., 2019) and 20-year (Fett et al., 2020) follow-up studies of younger, first-episode samples. Finally, the Vocabulary score, indexing verbal IQ, deteriorated across 20 years. Fett et al. (2020) reported a similar result.

We interpret these results as carefully suggestive of an accelerated ageing process in the verbal function domain. In particular, impairment in crystallized intelligence across time is usually a powerful indication of accelerated ageing, as in the general population, Vocabulary test scores tend to improve with age (Schaie, 1994). The acceleration process has been defined as neurodegenerative and linked to deterioration in grey and white matter in SSD patients (Stone et al., 2022). The reason for our caution, however, is the lack of a healthy comparison group that would enable us to confirm the presence of accelerated ageing. A further reason to be careful when interpreting our results is that we lack the statistical power to control for factors that may impact neurocognitive function, such as baseline education level (Starzer et al., 2024). Moreover, duration of illness and the age at illness debut may be relevant for neurocognitive and general function levels (Jonas et al., 2022). Early-onset psychosis is regarded as a more severe form of illness than later-onset psychosis (Chen et al., 2018; Köhler et al., 2007), and it is possible that those of our older participants with a relatively recent onset of illness would present with more preserved neurocognition throughout the study period.

Notably, the overall picture of our results is one of relative stability of neurocognitive function across 20 years. Stability in most domains in large groups of participants has also been found by others (Starzer et al., 2024; Flaaten et al., 2022; Rund et al., 2016; Zanelli et al., 2019). This suggests that the cognitive prospects of SSD patients as they age are relatively positive. However, the specific decline in verbal functions is cause for concern. Deficits of the verbal domain have been linked to long-term poorer symptomatic and social function as well as recurrent illness episodes in SSD patients (Cuesta et al., 2022). The clinical implication is that our participants may be in need of extra care and rehabilitation efforts.

### 4.1 Strengths and limitations

The major strengths of this study are the community sample of a naturalistic group of patients and our comprehensive neurocognitive test battery. A major limitation is our low N, which impacts the generalizability of our data in two important ways in addition to those already mentioned: First, we do not have sufficient statistical power to stratify the patients into different trajectories of stability or change and compare the demographic and clinical profiles of these subgroups (Starzer et al., 2024). Second, the small sample renders our interpretations of stability and change uncertain. However, the small effect sizes in time differences that show stability and the large effect sizes in the differences that show change permit us to be reasonably confident that we have detected true differences and not statistical artifacts. Third, we had access to insufficient information about medication treatment and were unable to control for the possible influence of types and doses of antipsychotics on neurocognitive test scores. Relatedly, assessment of psychosis symptoms with the PANSS was performed only at endline, preventing us to present a more detailed clinical picture across time. A fourth limitation is that we had no tests of visual learning and memory or social cognition in our battery. Fifth, intellectual function was indexed by only one test. Therefore, we do not possess the full picture of cognitive function in our participants. Finally, of the 500 SSD patients originally included in the CLIPS project, we had clinical or neurocognitive data for only 61 of them 20 years later. Attrition bias may have resulted in participants with poorer neurocognitive function to refrain from follow-up testing, as has been the case in similar studies (Starzer et al., 2024). We have previously reported that mortality at 20-year follow-up is predicted by cognitive impairment at baseline in a larger sample from the CLIPS project (Mohn et al., 2023). It is therefore likely that the oldest patients of the current study have more preserved cognitive function than those who display clear evidence of accelerated ageing.

## CRediT authorship contribution statement

**Christine Mohn:** Writing – review & editing, Formal analysis, Conceptualization. **Anna-Karin Olsson:** Writing – review & editing, Project administration, Investigation, Data curation, Conceptualization. **Maivor Olsson-Tall:** Writing – review & editing, Investigation. **Fredrik Hjärthag:** Writing – review & editing, Supervision, Conceptualization. **Iris van Dijk Härd:** Writing – review & editing, Investigation. **Lars Helldin:** Writing – review & editing, Supervision, Project administration, Methodology, Funding acquisition, Formal analysis, Data curation, Conceptualization.

## Funding

This study was funded by the Regional Health Authority, VG Region, Western Sweden, as part of their regular batch of funding for research and development activities. The funding body had no impact on the data analyses or manuscript preparation.

## Declaration of competing interest

The authors have nothing to declare.

## Appendix A Supplementary data

Supplementary tables Image 1
