# Supplementary material for: Neurocognitive function in schizophrenia spectrum disorders: A 20-year prospective study of a community sample
Source: Schizophr Res Cogn. 2025 Sep 30;43:100393. doi: 10.1016/j.scog.2025.100393 (PMC12512163; doi:10.1016/j.scog.2025.100393)
Supplement: Supplementary file 1 — Supplementary tables [file mmc1.docx]

Supplementary Table 1. Demographic and clinical information (N=61).

|  | **Baseline** | **20 years** |
| --- | --- | --- |
| **Sex** | 48 males (68.9)  19 females (31.1) |  |
| **Age at inclusion in study, years** | 42.9 (11.1), range 21-73 |  |
| **Age at first hospitalization, years** | 29.4 (10.5), range 15-63 |  |
| **Duration of treated illness, years** | 13.3 (10.2), range 0-39 |  |
| **In remission** | 37 (60.7) | 48 (78.7) # |
| **PANSS** |  |  |
| Positive |  | 10.5 (3.6) |
| Negative |  | 12.5 (5.1) |
| General |  | 22.3 (5.2) |
| **GAF** |  |  |
| Symptoms | 47.5 (9.4) | 56.1 (17.5) |
| Function | 50.4 (8.8) | 58.3 (15.4) |
| **Antipsychotic medication *** |  |  |
| Risperdal Consta |  | 4 (6.6) |
| Risperdal |  | 4 (6.6) |
| Clozapine |  | 11 (18.0) |
| Quetiapine |  | 4 (6.6) |
| Olanzapine |  | 6 (9.8) |
| Aripiprazole |  | 7 (11.5) |
| Paliperidone |  | 2 (3.3) |
| Ziprasidone |  | 1 (1.6) |

Numbers in mean (SD) or N (%). #: Missing data n=9 (14.8%). *: Missing data n=22 (36.1%).

Supplementary Table 2. Social and economic information (N=61)

|  | **Baseline** | **20 years** |
| --- | --- | --- |
| **Marital status** |  |  |
| Single (never partnered) | 35 (57.4) | 35 (57.4) |
| Divorced/widowed | 13 (21.3) | 5 (8.2) |
| Married/partnered | 12 (19.7) | 11 (18.0) |
| Missing data | 1 (1.6) | 9 (14.8) |
| **Housing status** |  |  |
| Long-term hospital/care | 2 (3.3) | 0 |
| Assisted living facility | 1 (1.6) | 2 (3.3) |
| Living with parents | 5 (8.2) | 2 (3.3) |
| Own residence | 51 (83.6) | 48 (78.7) |
| Missing data | 2 (3.3) | 9 (14.8) |
| **Education level #** |  |  |
| Elementary school | 19 (31.1) |  |
| High school | 28 (45.9) |  |
| College level | 13 (21.3) |  |
| Missing data | 1 (1.6) |  |
| **Employment status #** |  |  |
| Full-time work | 23 (37.7) |  |
| Part-time work | 3 (4.9) |  |
| Student | 4 (6.6) |  |
| Volunteer work | 1 (1.6) |  |
| Supported employment | 0 |  |
| Age/disability pension | 1 (1.6) |  |
| Never worked/unknown | 1 (1.6) |  |
| Missing data | 26 (42.6) |  |

Numbers in N (%). #: Highest level of education or employment before illness onset.

Supplementary Table 3. Baseline demographic and clinical information for participants with baseline (N=20) or endline (N=13) neurocognitive tests.

|  | **Baseline only** | **20 years only** |
| --- | --- | --- |
| **Sex** | 14 males (70.0)  6 females (30.0) | 8 males (61.5)  5 females (38.5) |
| **Age at inclusion in study, years** | 45.6 (10.4), range 26-60 | 42.0 (12.8), 21-63 |
| **Age at first hospitalization, years** | 30.2 (10.7), range 15-53 | 26.3 (8.2), 19-49 |
| **Duration of treated illness, years** | 14.4 (10.4), range 3-39 | 13.0 (10.4), 2-38 |
| **In remission** | 8 (40.0) | 6 (46.2) |
| **Sub-diagnosis** |  |  |
| Schizophrenia | 12 (60.0) | 10 (76.9) |
| Schizoaffective disorder | 7 (35.0) | 1 (7.7) |
| Delusional disorder | 1 (5.0) | 2 (15.4) |
| **GAF** |  |  |
| Symptoms | 47.4 (9.9) | 49.8 (13.6) |
| Function | 49.3 (8.9) | 50.4 (12.5) |

Numbers in mean (SD) or N (%).

Supplementary Table 4. Neurocognitive function in raw scores for baseline (N=20) or endline (N=13).

| **Cognitive domain** | **Baseline only** | **20 years only** |
| --- | --- | --- |
| *Processing speed*  TMT-A | 64.6 (63.7) | 65.4 (36.1) |
| *Cognitive flexibility*  TMT-B | 137.6 (83.7) | 205.1 (127.3) |
| *Immediate memory*  RAVLT-1 | 3.8 (1.8) | 3.2 (1.2) |
| *Short-term memory*  RAVLT 1-5 sum | 34.8 (11.2) | 29.0 (10.4) |
| *Retention memory*  RAVLT-7 | 6.0 (2.5) | 5.6 (3.1) |
| *Working memory*  LNS | 9.2 (2.6) | 6.3 (4.3) |
| *Executive function*  WCST Trials | 117.6 (20.4) | 120.5 (13.4) |
| WCST Total errors | 56.2 (29.3) | 51.3 (22.7) |
| WCST Perseverative  responses | 46.9 (40.5) | 38.3 (33.0) |
| WCST Perseverative  errors | 37.9 (29.7) | 30.4 (23.3) |
| WCST Completed  categories | 2.8 (2.3) | 3.1 (2.3) |
| *Crystallized intelligence*  WAIS Vocabulary | 40.9 (10.7) | 29.5 (15.0) |

Numbers in mean (SD).
